# In-Network Computation of the Optimal Weighting Matrix for Distributed Consensus on Wireless Sensor Networks

**DOI:** 10.3390/s17081702

**Published:** 2017-07-25

**Authors:** Xabier Insausti, Jesús Gutiérrez-Gutiérrez, Marta Zárraga-Rodríguez, Pedro M. Crespo

**Affiliations:** Department of Biomedical Engineering and Sciences, Tecnun, University of Navarra, Manuel Lardizábal 13, 20018 San Sebastián, Spain; jgutierrez@tecnun.es (J.G.-G.); mzarraga@tecnun.es (M.Z.-R.); pcrespo@tecnun.es (P.M.C.)

**Keywords:** consensus, distributed computation, networks

## Abstract

In a network, a distributed consensus algorithm is fully characterized by its weighting matrix. Although there exist numerical methods for obtaining the optimal weighting matrix, we have not found an in-network implementation of any of these methods that works for all network topologies. In this paper, we propose an in-network algorithm for finding such an optimal weighting matrix.

## 1. Introduction

A sensor is a device capable of measuring a certain physical property. Normally, in a wireless sensor network (WSN), each sensor or node can transmit and receive data wirelessly, and it has the ability of performing multiple tasks, which are usually based on simple mathematical operations such as additions and multiplications. Moreover, the sensors within a WSN are usually powered with batteries, leading to very limited energy resources.

For most tasks, it is required that each sensor computes a target value that depends on the values measured by other sensors of the WSN. Commonly, a WSN has a central entity, known as the central node, which collects the values measured by all the sensors, computes the target values, and sends each target value to the corresponding sensor. This strategy is known as centralized computation.

The main disadvantage of the centralized computation strategy is that it is extremely energy inefficient from the transmission point of view because, when a sensor is far away from the central node, it has to consume disproportionate amounts of energy, with respect to the energy provided by its battery, in order to transmit its measured value to the central node. An alternative strategy to overcome the energy inefficiency of the centralized computation is the distributed or in-network computation strategy. In distributed computation, which is a cooperative strategy, each sensor computes its target value by interchanging information with its neighbouring sensors.

In many recent signal processing applications of distributed computations (e.g., [1,2,3,4]), the average needs to be computed (i.e., each sensor seeks the arithmetic mean of the values measured by all the sensors of the WSN). The problem of obtaining that average in all the sensors of the WSN by using the distributed computation strategy is known as the distributed averaging problem, or the distributed average consensus problem. Moreover, the problem of obtaining the same value in all the sensors of the WSN by using the distributed computation strategy is known as the distributed consensus problem (see, for example, [5] for a review on this subject).

A common approach for solving the distributed averaging problem is to use a synchronous linear iterative algorithm that is characterized by a matrix, which is called the weighting matrix. A well-known problem related to this topic is that of finding a symmetric weighting matrix that achieves consensus as fast as possible. This is the problem of finding the fastest symmetric distributed linear averaging (FSDLA) algorithm.

The FSDLA problem was solved in [6]. Specifically, in [6], the authors proved that solving the FSDLA problem is equivalent to solving a semidefinite program, and they used the subgradient method for efficiently solving such a problem to obtain the corresponding weighting matrix. Unfortunately, solving the FSDLA problem this way requires a central entity with full knowledge of the entire network. This central entity has to solve the FSDLA problem and then communicate the solution to each node of the network. This process has to be repeated each time the network topology changes due to, for example, a node failing, a node being added or removed (plug-and-play networks), or a node changing its location.

Moreover, WSNs may not have a central entity to compute the optimal weighting matrix. This paper proposes, for those networks without a central entity, an in-network algorithm for finding the optimal weighting matrix.

It is worth mentioning that in the literature, one can find other in-network algorithms that solve the FSDLA problem in a distributed way. In particular, in [7], the authors present an in-network algorithm that computes the fastest symmetric weighting matrix, but only with positive weights. As will be made more explicit in the next section, this matrix is not a solution of the FSDLA problem in general, as the latter might contain negative weights.

In [8], the FSDLA problem is solved in a centralized way when the communication among nodes is noisy. Closed-form expressions for the optimal weights for certain network topologies (paths, cycles, grids, stars, and hypercubes) are also provided. However, unless the considered network topology is one of these five, an in-network solution to the FSDLA is not provided.

Finally, in [9], an in-network algorithm for solving the FSDLA problem is provided. However, as the authors claim, the algorithm breaks down when the second- and third-largest eigenvalues of the weighting matrix become similar or equal.

Unlike the approaches found in the literature, the in-network algorithm presented in this paper is proved to always converge to the solution of the FSDLA problem, irrespective of the considered network topology.

## 2. Preliminaries

### 2.1. The Distributed Average Consensus Problem

We consider a network composed of *n* nodes. The network can be viewed as an undirected graph G=(V,E), where V={1,2,…,n} is the set of nodes and E is the set of edges. An edge e={i,j}∈E means that nodes i,j∈V are connected and can therefore interchange information. Conversely, if {i,j}∉E, this means that nodes i,j∈V are not connected and cannot interchange information. We let *K* be the cardinal of E, i.e., *K* is the number of edges in the graph *G*. For simplicity, we enumerate the edges in the graph *G* as $E=\{e_{1},e_{2},\ldots ,e_{K}\}$, where $e_{k}=\left\{i_{k},j_{k}\right\}$ for all k∈{1,2,…,K}.

We assume that each node i∈V has an initial value $x_{i}\left(0\right)\in R$, where R denotes the set of (finite) real numbers. Accordingly, in this paper, $R^{m\times n}$ denotes the set of m×n real matrices. We consider that all the nodes are interested in obtaining the arithmetic mean (average) $x_{\mathrm{ave}}$ of the initial values of the nodes, that is,

$$x_{\mathrm{ave}}:=\frac{1}{n}\sum_{i=1}^{n}x_{i}\left(0\right),$$

using a distributed algorithm. This problem is commonly known as the distributed averaging problem, or the distributed average consensus problem.

The approach that will be considered here for solving the distributed averaging problem is to use a linear iterative algorithm of the form

(1) $$x_{i}\left(t+1\right)=w_{i,i}x_{i}\left(t\right)+\sum_{j\in V:\;\{i,j\}\in E}w_{i,j}x_{j}\left(t\right),\;i\in V$$

where time *t* is assumed to be discrete (namely, t∈{0,1,2,…}) and $w_{i,j}\in R$ are the weights that need to be set so that

(2) $$\lim_{t\to \infty }x_{i}\left(t\right)=x_{\mathrm{ave}}$$

for all i∈V and for all $x_{1}\left(0\right),x_{2}\left(0\right),\ldots ,x_{n}\left(0\right)\in R$. From the point of view of communication protocols, there exist efficient ways of implementing synchronous consensus algorithms of the form of Equation (1) (e.g., [10]).

We observe that Equation (1) can be written in matrix form as

(3) x(t+1)=Wx(t)

where $x\left(t\right)={\left(x_{1}\left(t\right),x_{2}\left(t\right),\ldots ,x_{n}\left(t\right)\right)}^{⊤}\in R^{n\times 1}$, and $W\in R^{n\times n}$ is called the *weighting matrix*, which is such that its entry at the *i*th row and *j*th column, ${\left[W\right]}_{i,j}$, is given by

(4) $${\left[W\right]}_{i,j}=\left\{\begin{array}{cc} 0 & \mathrm{if}\;i\neq j\;\mathrm{and}\;\{i,j\}\notin E,\\ w_{i,j} & \mathrm{otherwise}. \end{array}\right.\;i,j\in \left\{1,2,\ldots ,n\right\}$$

Therefore, Equation (2) can be rewritten as

(5) $$\lim_{t\to \infty }W^{t}=P_{n}$$

where $P_{n}:=\frac{1}{n}1_{n}1_{n}^{⊤}$, and $1_{n}$ is the n×1 matrix of ones.

We only consider algorithms of the form of Equation (3), for which the weighting matrix W is symmetric. If W is symmetric, it is shown in [6] (Theorem 1) that Equation (5) holds if and only if $W1_{n}=1_{n}$ and $∥W-P_{n}{∥}_{2}<1$, where ${∥\cdot ∥}_{2}$ denotes the spectral norm. For the reader’s convenience, we here recall that if $A\in R^{n\times n}$ is symmetric, then ${∥A∥}_{2}=\left|\lambda_{1}\left(A\right)\right|$, where $\lambda_{l}\left(A\right)$, l∈{1,2,…,n}, denote the eigenvalues of A, which, in this paper, are arranged such that $|\lambda_{1}\left(A\right)|\geq |\lambda_{2}\left(A\right)|\geq \ldots \geq |\lambda_{n}\left(A\right)|$ (e.g., [11] (pp. 350, 603)).

We observe that Equation (10) can be computed in a distributed way if each node i∈V is able to know $y_{i}$. The following result provides a means of computing such a unit eigenvector y of W(w) in a distributed way.

### 2.2. Considered Minimization Problem: FSDLA Problem

We denote with W(G) the set of all the n×n real symmetric matrices that satisfy Equation (4) and $W1_{n}=1_{n}$ simultaneously, that is,

$$\begin{array}{cc} W\left(G\right):=\{W\in R^{n\times n},\; & {\left[W\right]}_{i,j}=0\;\mathrm{if}\;i\neq j\;\mathrm{and}\;\left\{i,j\right\}\notin E,\\ & W=W^{⊤},\;W1_{n}=1_{n}\}. \end{array}$$

In [6], the convergence time of an algorithm of the form of Equation (3) with symmetric weighting matrix W is defined as

(6) $$\tau \left(W\right):=\frac{-1}{\log{∥W-P_{n}∥}_{2}}$$

This convergence time is a mathematical measure of the convergence speed of the algorithm.

According to the previous, we call the FSDLA problem to find a weighting matrix $W_{\mathrm{opt}}\in W\left(G\right)$ such that

(7) $$∥W_{\mathrm{opt}}-P_{n}{∥}_{2}\leq {∥W-P_{n}∥}_{2}\;\forall W\in W\left(G\right)$$

We observe that in this definition the meaning of *fastest* is in terms of convergence time.

It is shown in [6] that the FSDLA problem of Equation (7) is a constrained convex minimization problem that can be efficiently solved. In fact, in [6], it is shown that the FSDLA problem of Equation (7) can be expressed as a semidefinite program, and semidefinite programs can be efficiently solved [12]. However, to the best of our knowledge, there are yet no approaches for solving this FSDLA problem in a distributed (in-network) way. The contribution of this paper is to solve the FSDLA problem of Equation (7) in a distributed way. To do so, we develop a distributed subgradient method.

Finally, it should be mentioned that in [7], the authors solved, in a distributed way, a related problem: they find the fastest mixing Markov chain (FMMC). The FMMC problem is devoted to finding a matrix $W_{\mathrm{opt}}^{+}\in W\left(G\right)\cap \left\{W\in R^{n\times n}:\;{\left[W\right]}_{i,j}\geq 0,\;\forall i,j\in \left\{1,\ldots ,n\right\}\right\}$ such that $∥W_{\mathrm{opt}}^{+}-P_{n}{∥}_{2}\leq {∥W-P_{n}∥}_{2}$ for all $W\in W\left(G\right)\cap \{W\in R^{n\times n}:\;{\left[W\right]}_{i,j}\geq 0,\;\forall i,j\in \left\{1,\ldots ,n\right\}\}$. We observe that $∥W_{\mathrm{opt}}-P_{n}{∥}_{2}\leq {∥W_{\mathrm{opt}}^{+}-P_{n}∥}_{2}$, i.e., the solution of the FSDLA problem is faster than, or is at least as fast as, the solution of the FMMC problem.

### 2.3. FSDLA as an Unconstrained Convex Minimization Problem

In order to use a distributed subgradient method (the classical reference on subgradient methods is [13]), we first need to convert the FSDLA problem into an unconstrained convex minimization problem. We observe that if W∈W(G), it is clear that W depends on $w_{e_{k}}:=w_{i_{k},j_{k}}$ for all k∈{1,2,…,K}. We notice that $w_{e_{k}}$ is well defined because W is symmetric. In fact, as it was stated in [6], given the vector $W={\left(w_{e_{1}},w_{e_{2}},\ldots ,w_{e_{K}}\right)}^{⊤}\in R^{K\times 1}$, there exists a unique W∈W(G) such that ${\left[W\right]}_{i_{k},j_{k}}=w_{e_{k}}$ for all k∈{1,2,…,K}, namely

(8) $$W\left(w\right)=I_{n}+\sum_{k=1}^{K}w_{e_{k}}A_{k}$$

where $I_{n}$ is the n×n identity matrix and $A_{k}\in R^{n\times n}$ is defined as

$${\left[A_{k}\right]}_{i,j}:=\left\{\begin{array}{cc} 1 & \left\{i,j\right\}=\left\{i_{k},j_{k}\right\},\\ -1 & i=j=i_{k}\;\mathrm{or}\;i=j=j_{k},\\ 0 & \mathrm{otherwise}. \end{array}\right.\;\forall k\in \left\{1,2,\ldots ,K\right\}$$

In other words, the function $W:R^{K\times 1}\mapsto W\left(G\right)$ defined in Equation (8) is a bijection. We define the function $f:\;R^{K\times 1}\mapsto \left[0,\infty \right)$ as $f\left(w\right):={∥W\left(w\right)-P_{n}∥}_{2}$. We observe that the FSDLA problem of Equation (7) can now be expressed as an unconstrained minimization of the function *f*.

In the sequel, we denote with $\hat{w}$ a solution of the FSDLA problem, that is,

$$f\left(\hat{w}\right)\leq f\left(w\right)\;\forall w\in R^{K\times 1}.$$

It is easy to show that *f* has a bounded set of minimum points $\hat{w}$. In the sequel, we will refer to the function *f* as the cost function of the FSDLA problem. We finish the section with Lemma 1 which will be useful in the derivation of the algorithm.

**Lemma** **1.** *If $w\in R^{K\times 1}$, then*

$$f\left(w\right)=\left\{\begin{array}{cc} |\lambda_{1}\left(W\left(w\right)\right)| & if\;|\lambda_{2}\left(W\left(w\right)\right)|\geq 1,\\ |\lambda_{2}\left(W\left(w\right)\right)| & if\;|\lambda_{2}\left(W\left(w\right)\right)|<1. \end{array}\right.$$

**Proof.**  Observe that as W=W(w) is symmetric and $W1_{n}=1_{n}$, there exists an eigenvalue decomposition of W, $W=U{\mathrm{diag}}_{n}\left(1,\lambda_{2}\left(W\right),\ldots ,\lambda_{n}\left(W\right)\right)U^{⊤}$, where U is a real n×n orthogonal matrix such that ${\left[U\right]}_{i,1}=\frac{1}{\sqrt{n}}$ for all i∈{1,2,…,n} and $|\lambda_{2}\left(W\right)|\geq |\lambda_{3}\left(W\right)|\geq \ldots \geq |\lambda_{n}\left(W\right)|$. As $P_{n}=U{\mathrm{diag}}_{n}\left(1,0,\ldots ,0\right)U^{⊤}$, we have

$$\begin{array}{cc} f(w) & =∥W-P_{n}{∥}_{2}={∥U{\mathrm{diag}}_{n}\left(0,\lambda_{2}\left(W\right),\ldots ,\lambda_{n}\left(W\right)\right)U^{⊤}∥}_{2}\\ & =∥{\mathrm{diag}}_{n}\left(0,\lambda_{2}\left(W\right),\ldots ,\lambda_{n}\left(W\right)\right){∥}_{2}=\left|\lambda_{2}\left(W\right)\right|. \end{array}$$

☐

## 3. Algorithm for the In-Network Solution of the FSDLA Problem

We here derive the algorithm that solves the FSDLA problem in a distributed way (Algorithm 1). To this end, we assume that *n* is known by all the nodes of the network. The task of counting nodes can be performed in a distributed way (see [14]). The algorithm is a distributed implementation of a subgradient method. More specifically, each pair of nodes $\left\{i_{k},j_{k}\right\}$ will update their weight $w_{i_{k},j_{k}}$ according to the following iterative formula:

(9) $$w_{p+1}=w_{p}-\eta_{p+1}\;\tilde{\nabla }f\left(w_{p}\right)$$

where $w_{p}\in R^{K\times 1}$ is the vector of weights at the *p*th step, $\eta_{p}\in R$ is the stepsize, and $\tilde{\nabla }f\left(w\right)$ is a subgradient of *f* at w. We recall here that a vector $\tilde{\nabla }f\left(w\right)\in R^{K\times 1}$ is a subgradient of $f:\;R^{K\times 1}\mapsto R$ at $w\in R^{K\times 1}$ if $f\left(v\right)\geq f\left(w\right)+{\left(\tilde{\nabla }f\left(w\right)\right)}^{⊤}\left(v-w\right)$ for all $v\in R^{K\times 1}$.

**Theorem** **1.** *If $w\in R^{K\times 1}$ such that 0<f(w)<1, and $y={\left(y_{1},y_{2},\ldots ,y_{n}\right)}^{⊤}\in R^{n\times 1}$ is such that ∥y∥=1 and $W\left(w\right)y={\left(-1\right)}^{s}\left|\lambda_{2}\left(W\left(w\right)\right)\right|y$ for some s∈{1,2}, then a subgradient of f at w is*

(10) $$\tilde{\nabla }f\left(w\right)={\left(-1\right)}^{s+1}\left(\begin{array}{c} {\left(y_{i_{1}}-y_{j_{1}}\right)}^{2}\\ \vdots \\ {\left(y_{i_{K}}-y_{j_{K}}\right)}^{2} \end{array}\right)$$

We observe that Equation (10) can be computed in a distributed way if each node i∈V is able to know $y_{i}$. The following result provides a means of computing such a unit eigenvector y of W(w) in a distributed way.

The rest of the section is devoted to proving that Equation (9) can be computed in a distributed way (Theorems 1–3), and to proving that Equation (9) actually converges to $\hat{w}$ (Theorem 4).

In order to compute Equation (9) in a distributed way, we need to compute a subgradient of *f* in a distributed way. With this in mind, we review a result given in [6].

**Theorem** **2.** *If* $W\in R^{K\times 1}$ *is such that* 0<f(w)<1, *then for all* $x\left(0\right)\in R^{n\times 1}$,

(11) $$W\left(w\right)y_{s}={\left(-1\right)}^{s}\left|\lambda_{2}\left(W\left(w\right)\right)\right|y_{s}\;s\in \left\{1,2\right\}$$

*where*

(12) $$y_{s}:=\lim_{t\to \infty }\left(\frac{x(t)-x(t-2)}{{\left({\left(-1\right)}^{s}f\left(w\right)\right)}^{t}}+\frac{x(t-1)-x(t-3)}{{\left({\left(-1\right)}^{s}f\left(w\right)\right)}^{t-1}}\right)$$

*and* $x\left(t\right)={\left(W(w)\right)}^{t}x\left(0\right)$ *for all* t∈{0,1,2,…}. *Furthermore, given* s∈{1,2}, *for almost every* $x\left(0\right)\in R^{n\times 1}$, *the following assertions are equivalent*:

*(a)*  $y_{s}\neq 0_{n\times 1}$, *where* $0_{n\times 1}$ *is the* n×1 *zero matrix*.
*(b)*  ${\left(-1\right)}^{s}\left|\lambda_{2}\left(W\left(w\right)\right)\right|$ *is an eigenvalue of* W(w).

**Algorithm 1** In-network solution of the FSDLA problem.
1:p←02:**for all** pair of nodes $e_{k}=\left\{i_{k},j_{k}\right\}$ **do**3: ${\left[w_{0}\right]}_{k}\leftarrow 1/\max\left(d_{i_{k}},d_{j_{k}}\right)$4:**end for**5:p←p+16:**for all** nodes i∈V **do**7: ${\left[x\right]}_{i}\leftarrow \mathrm{rand}\left(\right)$           ▹ An arbitrary value8: ${\left[\gamma_{1}\right]}_{i}\leftarrow {\left[{\mathrm{ave}}_{w_{p}}\left(x,t_{0}\right)\right]}_{i}-{\left[{\mathrm{ave}}_{w_{p}}\left(x,t_{0}-1\right)\right]}_{i}$9: ${\left[\gamma_{2}\right]}_{i}\leftarrow {\left[{\mathrm{ave}}_{w_{p}}\left(x,t_{0}-1\right)\right]}_{i}-{\left[{\mathrm{ave}}_{w_{p}}\left(x,t_{0}-2\right)\right]}_{i}$10: $f\left(w_{p}\right)={∥W\left(w_{p}\right)-P_{n}∥}_{2}\leftarrow \sqrt{\frac{{\left[{\mathrm{ave}}_{w_{p}}\left({\left({\left[\gamma_{1}\right]}_{1}^{2},\ldots ,{\left[\gamma_{1}\right]}_{n}^{2}\right)}^{⊤},t_{0}\right)\right]}_{i}}{{\left[{\mathrm{ave}}_{w_{p}}\left({\left({\left[\gamma_{2}\right]}_{1}^{2},\ldots ,{\left[\gamma_{2}\right]}_{n}^{2}\right)}^{⊤},t_{0}\right)\right]}_{i}}}$11: **if** $f(w_{p})\geq 1$ **then**12:  $w_{p}\leftarrow w_{p-1}$13: **end if**14: ${\left[y_{1}\right]}_{i}\leftarrow \frac{{\left[{\mathrm{ave}}_{w_{p}}\left(x,t_{0}\right)\right]}_{i}-{\left[{\mathrm{ave}}_{w_{p}}\left(x,t_{0}-2\right)\right]}_{i}}{{\left(-f\left(w_{p}\right)\right)}^{t_{0}}}+\frac{{\left[{\mathrm{ave}}_{w_{p}}\left(x,t_{0}-1\right)\right]}_{i}-{\left[{\mathrm{ave}}_{w_{p}}\left(x,t_{0}-3\right)\right]}_{i}}{{\left(-f\left(w_{p}\right)\right)}^{t_{0}-1}}$15: ${\left[y_{2}\right]}_{i}\leftarrow \frac{{\left[{\mathrm{ave}}_{w_{p}}\left(x,t_{0}\right)\right]}_{i}-{\left[{\mathrm{ave}}_{w_{p}}\left(x,t_{0}-2\right)\right]}_{i}}{{\left(f\left(w_{p}\right)\right)}^{t_{0}}}+\frac{{\left[{\mathrm{ave}}_{w_{p}}\left(x,t_{0}-1\right)\right]}_{i}-{\left[{\mathrm{ave}}_{w_{p}}\left(x,t_{0}-3\right)\right]}_{i}}{{\left(f\left(w_{p}\right)\right)}^{t_{0}-1}}$16: $∥y_{1}∥\leftarrow \sqrt{{\left[{\mathrm{ave}}_{w_{p}}\left({\left({\left[y_{1}\right]}_{1}^{2},\ldots ,{\left[y_{1}\right]}_{n}^{2}\right)}^{⊤},t_{0}\right)\right]}_{i}}$17: **if** $∥y_{1}∥\neq 0$ **then**18:  ${\left[y\right]}_{i}\leftarrow {\left[y_{1}\right]}_{i}/∥y_{1}∥$19:  s←120: **else**21:  $∥y_{2}∥\leftarrow \sqrt{{\left[{\mathrm{ave}}_{w_{p}}\left({\left({\left[y_{2}\right]}_{1}^{2},\ldots ,{\left[y_{2}\right]}_{n}^{2}\right)}^{⊤},t_{0}\right)\right]}_{i}}$22:  ${\left[y\right]}_{i}\leftarrow {\left[y_{2}\right]}_{i}/∥y_{2}∥$23:  s←224: **end if**25:**end for**26:**for all** pair of nodes $e_{k}=\left\{i_{k},j_{k}\right\}$ **do**27: ${\left[\tilde{\nabla }f\left(w_{p}\right)\right]}_{k}\leftarrow {\left(-1\right)}^{s+1}{\left({\left[y\right]}_{i_{k}}-{\left[y\right]}_{j_{k}}\right)}^{2}$28: ${\left[w_{p}\right]}_{k}\leftarrow {\left[w_{p-1}\right]}_{k}-\beta_{p}{\left[\tilde{\nabla }f\left(w_{p}\right)\right]}_{k}$29:**end for**30:**if** $p<p_{\max}$ **go to** 5

**Proof.**  Let $W=W\left(w\right)=U{\mathrm{diag}}_{n}\left(1,\lambda_{2}\left(W\right),\ldots ,\lambda_{n}\left(W\right)\right)U^{⊤}$ be as in the proof of Lemma 1, with $U=\left(u_{1}|u_{2}\left|\ldots \right|u_{n}\right)$. Observe that $\lambda_{2}\left(W\right)\neq 0$, as $|\lambda_{2}\left(W\right)|=f\left(w\right)\neq 0$.

If ${\left(-1\right)}^{s-1}\left|\lambda_{2}\left(W\right)\right|$ is an eigenvalue of W for some s∈{1,2}, then we denote by $L_{s}$ its algebraic multiplicity. Otherwise we set $L_{s}=0$. From Lemma 1, $f\left(w\right)=|\lambda_{2}\left(W\right)|$ and consequently $L_{1}$ and $L_{2}$ cannot be simultaneously zero. Moreover, without loss of generality we can assume that $\lambda_{2}\left(W\right)\geq \ldots \geq \lambda_{L_{1}+L_{2}+1}\left(W\right)$.

Then, we have that

(13) $$\begin{array}{cc} x(t) & =W^{t}x\left(0\right)=W^{t}\left(\sum_{l=1}^{n}\alpha_{l}u_{l}\right)=\sum_{l=1}^{n}\alpha_{l}W^{t}u_{l}\\ & =\alpha_{1}W^{t}u_{1}+\sum_{l=2}^{L_{1}+1}\alpha_{l}W^{t}u_{l}+\sum_{l=L_{1}+2}^{L_{1}+L_{2}+1}\alpha_{l}W^{t}u_{l}+\sum_{l=L_{1}+L_{2}+2}^{n}\alpha_{l}W^{t}u_{l}\\ & =\alpha_{1}u_{1}+\sum_{l=2}^{L_{1}+1}\alpha_{l}|\lambda_{2}{\left(W\right)|}^{t}u_{l}+\sum_{l=L_{1}+2}^{L_{1}+L_{2}+1}\alpha_{l}{\left(-1\right)}^{t}{\left|\lambda_{2}\left(W\right)\right|}^{t}u_{l}+\sum_{l=L_{1}+L_{2}+2}^{n}\alpha_{l}\lambda_{l}{\left(W\right)}^{t}u_{l}\\ & =\alpha_{1}u_{1}+{\left|\lambda_{2}\left(W\right)\right|}^{t}\left(\sum_{l=2}^{L_{1}+1}\alpha_{l}u_{l}+{\left(-1\right)}^{t}\sum_{l=L_{1}+2}^{L_{1}+L_{2}+1}\alpha_{l}u_{l}\right)+\sum_{l=L_{1}+L_{2}+2}^{n}\alpha_{l}\lambda_{l}{\left(W\right)}^{t}u_{l}\\ & =\alpha_{1}u_{1}+{\left|\lambda_{2}\left(W\right)\right|}^{t}\left({\left(-1\right)}^{t}a_{1}+a_{2}\right)+r\left(t\right)\;\forall t\in \left\{0,1,2,\ldots \right\} \end{array}$$

where $\alpha_{l}={\left(x(0)\right)}^{⊤}u_{l}$ for all l∈{1,2,…,n},

$$a_{1}:=\sum_{l=L_{1}+2}^{L_{1}+L_{2}+1}\alpha_{l}u_{l},$$

$$a_{2}:=\sum_{l=2}^{L_{1}+1}\alpha_{l}u_{l},$$

and

$$r\left(t\right):=\sum_{l=L_{1}+L_{2}+2}^{n}\alpha_{l}\lambda_{l}{\left(W\right)}^{t}u_{l}.$$

Observe that

(14) $$Wa_{s}={\left(-1\right)}^{s}\left|\lambda_{2}\left(W\right)\right|a_{s}\;\forall s\in \left\{1,2\right\}$$

On the one hand, from Equation (13), we obtain

$$\begin{array}{cc} \frac{x(t)-x(t-2)}{|\lambda_{2}{\left(W\right)|}^{t}} & =\frac{|\lambda_{2}{\left(W\right)|}^{t}\left({\left(-1\right)}^{t}a_{1}+a_{2}\right)+r\left(t\right)}{|\lambda_{2}{\left(W\right)|}^{t}}-\frac{|\lambda_{2}{\left(W\right)|}^{t-2}\left({\left(-1\right)}^{t-2}a_{1}+a_{2}\right)-r\left(t-2\right)}{|\lambda_{2}{\left(W\right)|}^{t}}\\ & =\left(1-|\lambda_{2}{\left(W\right)|}^{-2}\right)\left({\left(-1\right)}^{t}a_{1}+a_{2}\right)+\frac{r(t)-r(t-2)}{|\lambda_{2}{\left(W\right)|}^{t}} \end{array}$$

for all t∈{2,3,…}. On the other hand, as $|\lambda_{l}\left(W\right)|<|\lambda_{2}\left(W\right)|$ for all $l\in \{L_{1}+L_{2}+2,\ldots ,n\}$, we have that

$$\begin{array}{c} \lim_{t\to \infty }\frac{r(t)}{|\lambda_{2}{\left(W\right)|}^{t}}=\lim_{t\to \infty }\sum_{l=L_{1}+L_{2}+2}^{n}\alpha_{l}\frac{\lambda_{l}{\left(W\right)}^{t}}{|\lambda_{2}{\left(W\right)|}^{t}}u_{l}=\lim_{t\to \infty }\sum_{l=L_{1}+L_{2}+2}^{n}\alpha_{l}{\left(\frac{\lambda_{l}\left(W\right)}{|\lambda_{2}\left(W\right)|}\right)}^{t}u_{l}=0_{n\times 1}. \end{array}$$

Consequently,

(15) $$\begin{array}{cc} y_{s} & =\lim_{t\to \infty }\left(\frac{x(t)-x(t-2)}{{\left({\left(-1\right)}^{s}\left|\lambda_{2}\left(W\right)\right|\right)}^{t}}+\frac{x(t-1)-x(t-3)}{{\left({\left(-1\right)}^{s}\left|\lambda_{2}\left(W\right)\right|\right)}^{t-1}}\right)\\ & =\lim_{t\to \infty }(\frac{1-|\lambda_{2}{\left(W\right)|}^{-2}}{{\left(-1\right)}^{st}}\left({\left(-1\right)}^{t}a_{1}+a_{2}+{\left(-1\right)}^{s}\left({\left(-1\right)}^{t-1}a_{1}+a_{2}\right)\right)\\ & \;+\frac{r(t)-r(t-2)}{{\left({\left(-1\right)}^{s}\left|\lambda_{2}\left(W\right)\right|\right)}^{t}}+\frac{r(t-1)-r(t-3)}{{\left({\left(-1\right)}^{s}\left|\lambda_{2}\left(W\right)\right|\right)}^{t-1}})\\ & =\lim_{t\to \infty }\left(\frac{1-|\lambda_{2}{\left(W\right)|}^{-2}}{{\left(-1\right)}^{st}}\left({\left(-1\right)}^{t}a_{1}+a_{2}+{\left(-1\right)}^{s}\left({\left(-1\right)}^{t-1}a_{1}+a_{2}\right)\right)\right)\\ & =2\left(1-|\lambda_{2}{\left(W\right)|}^{-2}\right)a_{s},\;s\in \left\{1,2\right\} \end{array}$$

Combining Equations (14) and (15), we obtain Equation (11).

From Equation (11), (a) implies (b) for all $x\left(0\right)\in R^{n\times 1}$.

As f(w)<1, from Lemma 1 and Equation (15), we have $y_{s}\neq 0_{n\times 1}$ if and only if $a_{s}\neq 0_{n\times 1}$. Consequently, if (b) holds, the set of x(0) such that $a_{s}=0_{n\times 1}$ is a vector space whose dimension is less than *n*; thus it has Lebesgue measure 0. Therefore, (a) and (b) are equivalent for almost every $x\left(0\right)\in R^{n\times 1}$. ☐

Theorem 2 implies that $∥y_{1}∥$ and $∥y_{2}∥$ cannot be zero simultaneously. Therefore, either $\frac{y_{1}}{∥y_{1}∥}$ or $\frac{y_{2}}{∥y_{2}∥}$ is the unit eigenvector required for computing Equation (10). We notice that the norm of a vector can be computed in a distributed way because it is the square root of *n* times the average of the squares of its entries. Consequently, we only need to know how to compute Equation (12) in a distributed way, or equivalently, how to compute the cost function *f* in a distributed way:

**Theorem** **3.** *If* $w\in R^{K\times 1}$ *such that* f(w)≠0, *then*

(16) $$f\left(w\right)=\lim_{t\to \infty }\frac{∥x(t)-x(t-1)∥}{∥x(t-1)-x(t-2)∥}$$

*for almost every* $x\left(0\right)\in R^{n\times 1}$, *where* $x\left(t\right)={\left(W(w)\right)}^{t}x\left(0\right)$ *for all* t∈{0,1,2,…}.

**Proof.**  Let $W=W\left(w\right)=U{\mathrm{diag}}_{n}\left(1,\lambda_{2}\left(W\right),\ldots ,\lambda_{n}\left(W\right)\right)U^{⊤}$ be as in the proof of Lemma 1, with $U=\left(u_{1}|u_{2}\left|\ldots \right|u_{n}\right)$. Then,

$$\begin{array}{cc} x(t) & =W^{t}x\left(0\right)=W^{t}\left(\sum_{l=1}^{n}\alpha_{l}u_{l}\right)=\sum_{l=1}^{n}\alpha_{l}W^{t}u_{l}\\ & =\alpha_{1}W^{t}u_{1}+\sum_{l=2}^{n}\alpha_{l}W^{t}u_{l}=\alpha_{1}u_{1}+\sum_{l=2}^{n}\alpha_{l}\lambda_{l}{\left(W\right)}^{t}u_{l} \end{array}$$

for all t∈{0,1,2,…}, where $\alpha_{l}={\left(x(0)\right)}^{⊤}u_{l}$ for all l∈{1,2,…,n}. Consider L∈{0,1,…,n−2} such that $|\lambda_{2}\left(W\right)|=|\lambda_{3}\left(W\right)|=\ldots =|\lambda_{2+L}\left(W\right)|$. Observe that $\lambda_{2}\left(W\right)\neq 0$, as $|\lambda_{2}\left(W\right)|=f\left(w\right)\neq 0$. Consequently, from the pythagorean theorem,

$$\begin{array}{cc} {∥x(t)-x(t-1)∥}^{2} & ={∥\sum_{l=2}^{n}\alpha_{l}\left(\lambda_{l}{\left(W\right)}^{t}-\lambda_{l}{\left(W\right)}^{t-1}\right)u_{l}∥}^{2}=\sum_{l=2}^{n}\alpha_{l}^{2}{\left(\lambda_{l}{\left(W\right)}^{t}-\lambda_{l}{\left(W\right)}^{t-1}\right)}^{2}\\ & =\sum_{l=2}^{n}\alpha_{l}^{2}{\left(\lambda_{l}\left(W\right)-1\right)}^{2}\lambda_{l}{\left(W\right)}^{2t-2}\\ & =\lambda_{2}{\left(W\right)}^{2t-2}\left(\sum_{l=2}^{L+2}\alpha_{l}^{2}{\left(\lambda_{l}\left(W\right)-1\right)}^{2}+\sum_{l=L+3}^{n}\alpha_{l}^{2}{\left(\lambda_{l}\left(W\right)-1\right)}^{2}{\left(\frac{\lambda_{l}\left(W\right)}{\lambda_{2}\left(W\right)}\right)}^{2t-2}\right) \end{array}$$

for all t∈{1,2,…}. Assume that $\sum_{l=2}^{L+2}\alpha_{l}^{2}\neq 0$, which holds for almost every $x\left(0\right)\in R^{n\times 1}$. As $|\lambda_{2}\left(W\right)|>|\lambda_{l}\left(W\right)|$ for all l∈{L+3,…,n}, we conclude that

$$\lim_{t\to \infty }\frac{∥x(t)-x(t-1)∥}{∥x(t-1)-x(t-2)∥}=|\lambda_{2}\left(W\right)|\sqrt{\frac{\sum_{l=2}^{L+2}\alpha_{l}^{2}{\left(\lambda_{l}\left(W\right)-1\right)}^{2}}{\sum_{l=2}^{L+2}\alpha_{l}^{2}{\left(\lambda_{l}\left(W\right)-1\right)}^{2}}}=\left|\lambda_{2}\left(W\right)\right|=f\left(w\right).$$

☐

We observe that Equation (16) can be computed in a distributed way because a norm can be computed in a distributed way. Moreover, we observe that the condition f(w)=0 holds if and only if $W=P_{n}$ (this is possible only if every pair of nodes of the network is connected, i.e., it is a fully-connected network; in this case, $W_{\mathrm{opt}}=P_{n}$ and consequently the FSDLA problem makes no sense). Therefore, for any non-fully-connected network, f(w)≠0.

At this point, we have shown that the iterative Equation (9) can be computed in a distributed way. It only remains to be shown that Equation (9) actually converges to $\hat{w}$:

**Theorem** **4.** *Consider* $w_{0}\in R^{K\times 1}$ *such that* $0<f(w_{0})<1$. *Let* $\left\{\eta_{p}\right\}$ *be a sequence of real numbers satisfying* $\lim_{p\to \infty }\eta_{p}=0$ *and* $\sum_{p=0}^{\infty }\eta_{p}=\infty$. *We also assume that*

(17) $$0<f\left(w_{p}\right)<1\;\forall p\in \left\{1,2,\ldots \right\}$$

*where* $w_{p}$ *is defined in Equation* (9). *Then*, $f\left(\hat{w}\right)={∥W_{\mathrm{opt}}-P_{n}∥}_{2}=\lim_{p\to \infty }f\left(w_{p}\right)$.

**Proof.**  Theorem 1 yields

$$\eta_{p}∥\tilde{\nabla }f\left(w_{p}\right)∥=\eta_{p}\sqrt{\sum_{k=1}^{K}{\left(y_{i_{k}}-y_{j_{k}}\right)}^{4}}\leq 4\sqrt{K}\max_{p\in \{0,1,2,\ldots \}}\eta_{p}.$$

Consequently, as *f* has a bounded set of minimum points, the result now follows from [13] (Theorem 2.4). ☐

We observe that the initial point $w_{0}$ in Theorem 4 can be taken, for instance, as that given by the Metropolis-Hastings algorithm (e.g., [8]). That is, if $w_{0}$ is that given by the Metropolis-Hastings algorithm, then ${\left[w_{0}\right]}_{k,1}=\frac{1}{\max\left(d_{i_{k}},d_{j_{k}}\right)}$ for all k∈{1,2,…,K}, where $d_{i}$ is the degree of node i∈V (i.e., the number of nodes to which node *i* is connected). Therefore, $w_{0}$ can be computed in a distributed way.

Table 1 relates Algorithm 1 with the theoretical aspects shown in this section.

**Remark** **1.** *As f is continuous, from every initial sequence of real numbers $\left\{\beta_{p}\right\}$ with $\lim_{p\to \infty }\beta_{p}=0$ and $\sum_{p=0}^{\infty }\beta_{p}=\infty$ (e.g. $\left\{\beta_{p}\right\}=\left\{1/\sqrt{p}\right\}$), a subsequence of stepsizes $\left\{\eta_{p}\right\}=\left\{\beta_{\sigma (p)}\right\}$ satisfying Equation* (17) *can be constructed.*

We finish the section by describing Algorithm 1. For ease of notation, we define

$${\mathrm{ave}}_{w}\left(x,t\right):={\left(W(w)\right)}^{t}x\;t\in \left\{0,1,2,\ldots \right\},\;x\in R^{n\times 1},$$

which is the *t*th iteration of Equation (1) and can clearly be computed in a distributed way. As for Algorithm 1, we fix $t_{0}$ to be the number of iterations of Equation (1) required for a desired precision. We observe that because the worst possible network topology is a path, if we set $t_{0}\geq \frac{\log\epsilon }{\log\cos(\pi /n)}$, then $∥{\mathrm{ave}}_{w}\left(x,t_{0}\right)-x_{\mathrm{ave}}1_{n}{∥}_{2}\leq \epsilon {∥x∥}_{2}$ (see [15]), and therefore $t_{0}$ can also be obtained in a distributed way.

## 4. Numerical Results

We here present the numerical results obtained using Algorithm 1 for two networks with n=16 nodes. The chosen starting point $w_{0}$ was that given by the Metropolis-Hastings algorithm [8], and the chosen initial sequence of stepsizes was $\left\{\beta_{p}\right\}=\left\{\frac{1}{\sqrt{p}}\right\}$ for all p∈{1,2,…}. Moreover, we took $t_{0}=250\approx \frac{\log10^{-2}}{\log\cos(\pi /16)}$.

**Figure 1:**
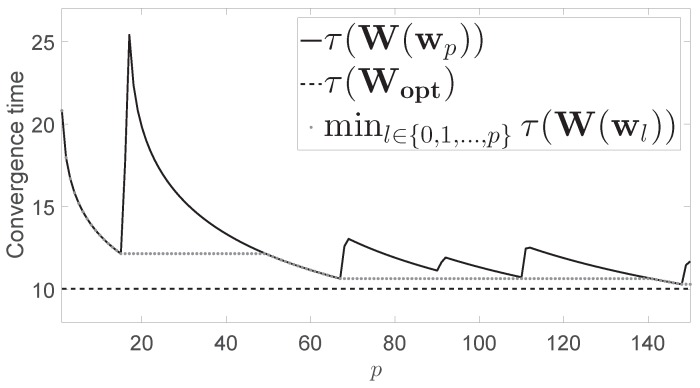
Numerical results for the graph of 16 nodes shown in Figure 2.

**Figure 2:**
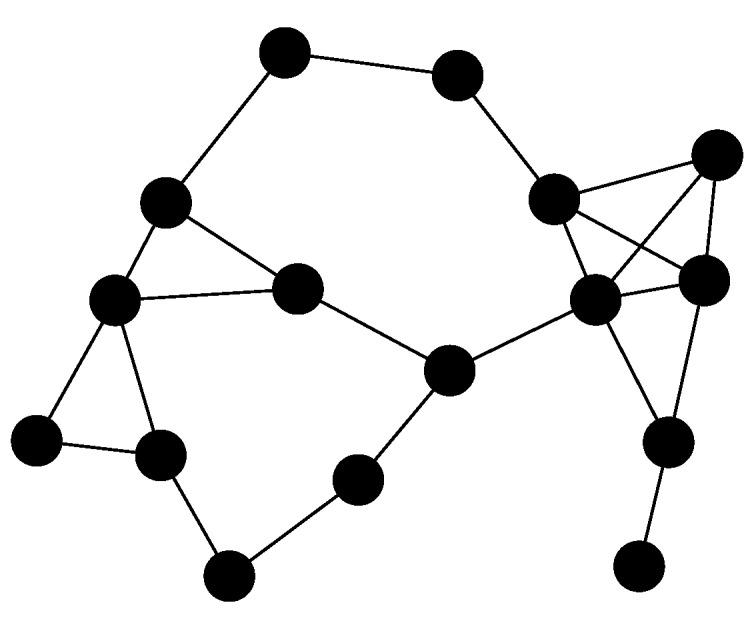
Graph with n=16 nodes considered in Figure 1.

Figure 1 shows the convergence time $\tau \left(W(w_{p})\right)$ for the network presented in Figure 2 (solid line). Figure 1 also shows $\tau (W_{\mathrm{opt}})=10.03$, which was obtained by using CVX, a package for specifying and solving convex programs in a centralized way [16,17] (dashed line). Finally, Figure 1 also shows the minimum value of $\tau \left(W(w_{p})\right)$ obtained up to step *p* (dotted line). For comparison purposes, we observe that the convergence time yielded by the Metropolis-Hastings algorithm was $\tau \left(W(w_{0})\right)=20.81$, while the minimum convergence time obtained after 150 iterations of our algorithm was 10.31.

**Figure 3:**
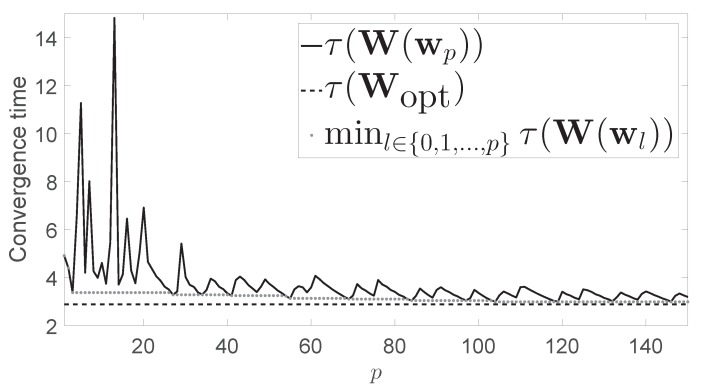
Numerical results for the grid of 16 nodes (4 rows and 4 columns).

Figure 3 is of the same type as Figure 1, but in this case, the considered network was a 4×4 grid. In this case, if the problem is optimally solved in a centralized way it yields $\tau (W_{\mathrm{opt}})=2.89$. The convergence time yielded by the Metropolis-Hastings algorithm was $\tau \left(W(w_{0})\right)=4.91$, while the minimum convergence time obtained after 150 iterations of our algorithm was 2.99.

We finish the section with a note on the number of exchanged messages (number of transmissions). For every iteration *p* of Algorithm 1, the number of exchanged messages per node was at most $5t_{0}$, divided as follows: $t_{0}$ message exchanges were required for lines 8 and 9, another $2t_{0}$ message exchanges were needed in line 10 (lines 14 and 15 did not require new message exchanges), and line 16 required another $t_{0}$ message exchanges. Finally, depending on the if-clause, another $t_{0}$ message exchanges were required in line 21. Therefore, the overall number of required transmissions per node was between $4p_{\max}t_{0}$ and $5p_{\max}t_{0}$.

## 5. Conclusions

In this paper we have provided an algorithm for the in-network computation of the optimal weighting matrix for distributed consensus. The algorithm can be viewed as an iterative repetition of, at most, five distributed consensus operations. Our algorithm is especially useful for networks that do not have a central entity and that change with time. In fact, if a network never changes with time (and its topology is known a priori), it seems easier to solve the FSDLA problem offline (in a centralized rather than a distributed way) using [6], and then pre-configuring the nodes with the obtained weights. However, if the network topology changes randomly with time (e.g., if sensors are added or removed) and there is no central entity, our algorithm would so far be the only way of obtaining the optimal solution to the FSDLA problem.

## Figures and Tables

**Table 1:** Explanation of Algorithm 1.

Lines	Description
2–4	Initialize with Metropolis-Hastings algorithm (Theorem 4)
7–10	Computation of the cost function *f* according to Theorem 3
11–13	Choose the correct subsequence according to Remark 1
14–15	Compute $y_{1}$ and $y_{2}$ as in Theorem 2
17–24	Obtain a unit eigenvector y from $y_{1}$ and $y_{2}$
27	Compute subgradient as in Theorem 1
28	Update as in Equation (9)
